# Dietary milk thistle meal supplementation modulates gut microbiota and improves performance and egg quality in late-phase laying hens

**DOI:** 10.3389/fmicb.2026.1925351

**Published:** 2026-08-12

**Authors:** Yanrong Long, Yuan Gao, Desheng Li, Rui Zhang, Xi Yang

**Affiliations:** 1 Department of Medical Technology, Guiyang Kangyang University, Guiyang, Guizhou, China; 2 College of Animal Science and Veterinary Medicine, Jinzhou Medical University, Jinzhou, China

**Keywords:** egg quality, excreta microbiota, laying hens, milk thistle meal, performance

## Abstract

The decline in production performance of late-phase laying hens is closely associated with gut microbiota dysbiosis. This study evaluated whether dietary milk thistle meal (MTM) could improve performance by modulating the gut microbiota. A total of 450 Jinghong 3.0 laying hens (55 weeks old) were randomly allocated to five dietary treatments: a basal diet or diets with 2.5, 5.0, 7.5, or 10.0% MTM, respectively. The experiment lasted 8 weeks. Overall, results indicated dose-dependent effects of MTM on hen performance. Specifically, both average egg weight and feed conversion ratio (FCR) showed significant linear and quadratic responses to MTM level (*P* < 0.05). Notably, during the trial, the MTM5.0 group exhibited the highest average egg weight among all groups and lower FCR than the MTM10.0 group (*P* < 0.05). Regarding egg quality, yolk color was significantly improved in groups receiving ≥ 5.0% MTM compared with that of the CON group (*P* < 0.05), while the Haugh unit was highest in the 5.0% MTM group (*P* < 0.05). Similarly, the apparent crude protein digestibility was also highest in the MTM5.0 group (*P* < 0.05). 16S rRNA sequencing of excreta samples revealed that MTM modulated specific microbial taxa in a dose-dependent manner, with the 5.0% inclusion level associated with the best performance. Specifically, the relative abundance of genus *Turicibacter* increased linearly with dietary MTM level (*P* < 0.05). Additionally, the relative abundance of the phylum *Bacteroidota* and genus *Bacteroides* showed significant quadratic trends (*P* < 0.05). Functional prediction (PICRUSt2) further revealed that MTM predominantly influenced amino acid, carbohydrate, and lipid metabolism pathways, with the most pronounced effects at the 2.5% dose. Collectively, dietary inclusion of 5.0% MTM improved production performance and egg quality in late-phase laying hens, potentially through maintenance of gut microbial homeostasis and enhanced nutrient utilization.

## Introduction

Globally, continuing population growth and rising living standards have boosted consumer demand for animal products (e.g., meat and eggs), which subsequently leads to severe challenges of feed resource shortages in the livestock industry (Ashayerizadeh et al., 2024; Madacussengua et al., 2024). Currently, poultry production heavily relies on conventional ingredients such as corn and soybean meal (Lei et al., 2018; Pope et al., 2023). However, the reliance on conventional feed ingredients results in competition with human food production for arable land, while the utilization of agricultural by-products as feed ingredients offers an effective strategy to mitigate this conflict (Van Zanten et al., 2018; Sandström et al., 2022). The implementation of such alternative feeding strategies, however, must address the specific challenges faced by production animals. For instance, in late-phase laying hens, aging and stress factors usually induce physiological decline, metabolic imbalance, reduced antioxidant capacity, impaired immune function, and disrupted gut microbiome, ultimately resulting in decreased production performance (Dai et al., 2022; Hu et al., 2025; Wei et al., 2025). Therefore, it would be judicious to select suitable agricultural by-products, which can simultaneously maintain or enhance production performance and improve egg quality, as alternative feeds for late-phase laying hens. Such an approach would not only alleviate feed resource shortages but also enhance the sustainability of the poultry industry.

Milk thistle (*Silybum marianum*) is a member of the Asteraceae family, exhibiting strong adaptability to various growing environments and a wide global distribution (Karkanis et al., 2011). In addition to its longstanding medicinal applications, milk thistle is now being explored as health products for humans and animals (Marceddu et al., 2022; Nehmi-Filho et al., 2022; Tedesco and Guerrini, 2023). Milk thistle meal (MTM), a byproduct obtained from the oil extraction of milk thistle seeds, is rich in protein and bioactive compounds such as silymarin, making it a promising unconventional feed resource. According to previous findings, MTM contains approximately 25% crude protein and is a good source of the limiting amino acid methionine (Lambo et al., 2024). These properties make it a promising alternative protein source. Furthermore, available evidence indicates that the total phenolic content (predominantly composed of the silymarin complex) of MTM is 470.64 mg gallic acid equivalent per gram of sample (Hashemi Jabali et al., 2018). Additionally, silymarin is the core component of pharmaceuticals and health supplements derived from milk thistle. Thus, the appreciable content of protein and bioactive compounds (especially silymarin) makes MTM a potential functional feed ingredient worthy of in-depth exploration.

Studies have shown that dietary silymarin supplementation enhances both the production performance and egg quality of laying hens, leading to increased egg production rate, greater egg weight, improved albumen quality, and a lower feed conversion ratio (Ahammad et al., 2024a; Guo et al., 2024; Khan et al., 2024). In addition to its benefits for laying hens, silymarin has been shown to enhance the growth performance, meat quality, and overall health of broilers by improving intestinal health, boosting antioxidant capacity, and promoting nutrient digestion and absorption (Jahanian et al., 2017; Jahanian et al., 2021; Shanmugam et al., 2022). Moreover, previous work has demonstrated that nanonized or lecithinized silymarin at a dosage of 200 mg/kg body weight effectively improved hepatic health in aged laying hens (Faryadi et al., 2024). Despite the well-established benefits of silymarin, research on the application of its source material, MTM, in layer diets remains limited. Dietary MTM supplementation has been shown to enhance health indicators, and hepatic health indices and oxidative stability were improved by the dietary combination of poultry oil and 30 g/kg of MTM (Gholamalian et al., 2022). In light of the above, although no studies have investigated the use of MTM in late-phase laying hens, the bioactive compounds in MTM (particularly silymarin) may offer a sustainable nutritional strategy to mitigate the decline in production performance during the late laying phase.

The gut microbiota plays a pivotal role in chicken health and productivity, with its composition directly linked to the host’s nutrient utilization, immune function, and overall gut health (Khan et al., 2020; Ricke et al., 2022; Zhang et al., 2022). Research on various animal and disease models has demonstrated that silymarin, the primary bioactive compound in MTM, functions in modulating the gut microbiota (Shen et al., 2019; Sun et al., 2023; Jin et al., 2024). Studies on the dietary application of silymarin in chickens have also demonstrated its modulatory effect on the gut microbiota (Shanmugam et al., 2022; Guo et al., 2024; Han et al., 2024). Consequently, MTM may sustain hen health and delay the decline in egg production during the late laying period by regulating intestinal microecological balance.

Furthermore, since MTM is a low-cost and underutilized agricultural processing by-product, incorporating it into poultry feed can reduce the poultry industry’s reliance on expensive conventional feed ingredients (e.g., corn and soybean meal). This strategy will not only enable the valorization of agricultural by-products but also improve the sustainability and economic viability of laying hen production systems. Nevertheless, the feasibility of dietary MTM application in late-phase laying hens, along with its optimal inclusion level and its impacts on production performance, egg quality, and excreta microbiota, has not been elucidated. Therefore, the current work aims to evaluate the effects of graded dietary MTM levels on the production performance, egg quality, and excreta microbiota in late-phase Jinghong 3.0 laying hens. We hypothesize that moderate MTM inclusion enhances nutrient utilization and promotes the enrichment of beneficial gut microbiota, thereby improving production performance and egg quality in late-phase laying hens. This study will provide new insights into exploring alternatives to conventional feed ingredients (e.g., corn and soybean meal) and enhancing the value of agricultural by-products, thereby supporting sustainable poultry production.

## Materials and methods

### Experimental design, animals, and rearing management

All procedures in this study were approved and supervised by the Animal Care and Use Committee of Jinzhou Medical University (Jinzhou, China). The feeding trial was carried out at Hongqiang Animal Husbandry Farm in Jinzhou City. A total of 450 Jinghong 3.0 laying hens (55 weeks of age) were randomly assigned to one of five dietary treatments. The experiment employed a completely randomized design with 5 replicates per treatment. Each replicate contained 18 hens housed in 6 cages, with 3 hens per cage. The replicate served as the experimental unit. Hens received a corn-soybean basal diet supplemented with 0% (CON), 2.5% (MTM2.5), 5.0% (MTM5.0), 7.5% (MTM7.5), and 10.0% (MTM10.0) MTM, respectively, according to a previous study (Hashemi Jabali et al., 2018). The MTM was included via equal-mass substitution with corn and soybean meal (1 kg MTM replaced 0.5 kg of corn and 0.5 kg of soybean meal; Table 1). The experiment consisted of a 10-day adaptation period followed by a 56-day formal experimental period.

**TABLE 1:** Composition and nutrient levels of diets (air-dry basis).

Items	Groups^1^				
	CON	MTM2.5	MTM5.0	MTM7.5	MTM10.0
Ingredients, %					
Milk thistle meal (MTM)^2^	0.00	2.50	5.00	7.50	10.00
Corn	63.40	62.15	60.90	59.65	58.40
Soybean meal	24.00	22.75	21.50	20.25	19.00
Limestone	8.00	8.00	8.00	8.00	8.00
Premix^3^	4.00	4.00	4.00	4.00	4.00
Soybean oil	0.60	0.60	0.60	0.60	0.60
Total	100	100	100	100	100
Nutrient levels					
Metabolizable energy, MJ/kg	11.49	11.45	11.41	11.37	11.33
Crude protein^4^, %	15.40	15.34	15.29	15.25	15.20
Calcium, %	3.81	3.81	3.82	3.80	3.81
Available phosphorus, %	0.35	0.35	0.35	0.35	0.35
Lysine, %	0.86	0.87	0.86	0.86	0.85
Methionine, %	0.42	0.41	0.41	0.40	0.41

^1^Groups: CON, basal diet without MTM; MTM2.5, diet with 2.5% MTM; MTM5.0, diet with 5.0% MTM; MTM7.5, diet with 7.5% MTM; MTM10.0, diet with 10.0% MTM.

^2^Analysed composition and nutrient levels of MTM (air-dry basis): Gross energy = 17.11 MJ/kg; Metabolizable energy = 8.86 MJ/kg; Dry matter = 91.95%; Crude protein = 23.61%; Crude fat = 2.08%; Crude fiber = 16.22%; Neutral detergent fiber = 38.56%; Acid detergent fiber = 22.73%; Ash = 5.32%; Calcium = 0.28%; Phosphorus = 0.68%.

^3^Premix provided the following per kilogram of diet: VA 10,000 IU, VD_3_ 3,200 IU, VE 40 IU, VK_3_ 1.2 mg, VB_1_ 2.4 mg, VB_2_ 8 mg, VB_6_ 4 mg, VB_12_ 0.032 mg, Biotin 0.2 mg, Nicotinic Acid 32 mg, Pantothenic Acid 8.8 mg, Folic Acid 1.6 mg, Choline Chloride 400 mg, Phytase 400 mg, Methionine 1,000 mg, Cu 6 mg, Fe 48 mg, Zn 84 mg, Mn 64 mg, I 1 mg, Se 0.2 mg.

^4^Crude protein were measured values, while other parameters were calculated.

The hens were housed in two-tiered battery cages. All hens had free access to water and were manually fed twice daily at 07:00 and 16:00. Eggs were collected once daily at 10:00 AM. Throughout the trial, a 16-h light schedule was maintained, and the ambient temperature within the housing room was maintained at 22–25°C.

MTM was kindly provided by Liaoning Kaiwei Biotechnology Co., Ltd. free of charge. The composition and nutrient levels of MTM (air-dry basis) are described in the legend below Table 1. The metabolizable energy of MTM in chickens was determined according to a previously established procedure (Hashemi Jabali et al., 2018). The composition and nutritional levels of experimental diets were formulated according to the Chinese agricultural standard *Feeding Standard of Chicken* (NY/T 33-2004) (China, Ministry of Agriculture of the People’s Republic of China, 2004; Table 1).

### Data collection and sample measurements

#### Production performance

During the experimental period, the number of eggs laid, total egg weight, and total feed intake per replicate were measured and recorded daily. Based on the data acquired above, the laying rate, average egg weight, average daily feed intake (ADFI), and feed conversion ratio (FCR, feed/egg, g/g) were calculated for each replicate.


Layingrate(%)

 =(total⁢number⁢of⁢eggs⁢laid/number⁢of⁢hens)×100%

Average⁢egg⁢weight⁢(g)

 =total⁢egg⁢weight/total⁢number⁢of⁢eggs⁢laid

Average⁢daily⁢feed⁢intake⁢(ADFI,g/d)

 =totalfeedintake/numberofhens

FeedconversionratioFCR,g/g)

 =total⁢feed⁢intake/total⁢egg⁢weight.

#### Egg quality

Egg quality was assessed using eggs collected from each replicate on the last two experimental days (days 55–56). Specifically, eggs from day 55 were stored at 4°C overnight and then pooled with fresh eggs from day 56. From each replicate, eggs falling within the overall median egg weight range determined from all treatment groups (approximately 60–62 g) were identified, and six were randomly selected for quality evaluation to minimize the confounding effect of egg size on the measurement of egg quality parameters. The short and long axes of eggs were measured using digital calipers to calculate the egg shape index (accuracy ± 0.01 mm). Eggshell strength was determined using an eggshell strength tester (RH-DQ200, Guangzhou Runhu Instrument CO., Ltd., China). An automated egg analyzer (EA-01, ORKA, Israel) was used to measure egg weight, albumen height, yolk color, and Haugh unit. Yolk weight, albumen weight, and shell weight were weighed using an analytical balance. Egg shape index, yolk ratio, albumen ratio and shell ratio were calculated based on the measured parameters.


$$\begin{array}{c} \begin{array}{c} \text{Egg weight (g)}=\text{(total egg weight from six eggs} \end{array}\\ \text{ per replicate})/\text{6}\\ \text{Egg shape index}=\text{long axis}/\text{short axis}\\ \text{Yolk ratio (\%) = (yolk weight}/\text{egg weight})\times 100\%\\ \text{Albumen ratio (\%) = (albumen weight}/\text{egg weight})\times 100\text{\%}\\ \text{Shell ratio (\%) = (shell weight}/\text{egg weight})\times 10\text{0\%.} \end{array}$$

#### Nutrient contents and apparent digestibility

For determination of apparent nutrient digestibility, 6 hens (1 hen per cage) were randomly selected from each replicate at the end of the trial and allocated to cages equipped with excreta collection trays. Feed and excreta were collected for subsequent nutrient content analysis.

On day 56, feed samples from each replicate were collected using the quartering method described in the Chinese National Standard *Animal feed*—*Preparation of test samples* (GB/T 20195-2024) (China, Standardization Administration of the People’s Republic of China, 2024a). Briefly, a pre-collected feed sample from the metabolism trial diet was coarsely ground and then reduced by successive quartering. The ground sample was thoroughly mixed, piled into a cone, and flattened into a uniform circular layer, which was divided into four equal segments with a cross-shaped divider. Two opposite segments were discarded, and the remaining two were combined. This process was repeated until a subsample of approximately 500 g was obtained for laboratory analysis. During the excreta collection period, feed intake per replicate was recorded daily at 08:00 and 16:00. Actual consumption was calculated from the weight of feed offered and residues.

Excreta were collected according to a previously described method (Kang et al., 2018). A total of 72-h excreta collection was conducted using a marker-to-marker approach. The appearance of chromic oxide (0.3%, added at the start) and ferric oxide (0.3%, added at the end) in droppings signaled the commencement and termination of collection, respectively (Kang et al., 2018). Excreta from each cage were collected 2 times daily (at 12 h intervals). Precautions were taken during collection to prevent contamination from feathers, feed, and other extraneous materials. The samples were stored at −20°C until further processing. Before analysis, the excreta samples were oven-dried at 65°C to constant weight and then ground through a 1-mm sieve.

The gross energy of the diets and excreta samples was measured with a bomb calorimeter (Parr Instrument, United States) according to the Chinese National Standard *Determination of gross energy of feeds*—*Bomb calorimetric method* (GB/T 45104-2024) (China, Standardization Administration of the People’s Republic of China, 2024b). Crude protein was analyzed using the Kjeldahl method, and crude fat content was determined using Soxhlet extraction, according to the Chinese National Standards *Determination of crude protein in feed* (GB/T 6432-2018) (China, Standardization Administration of the People’s Republic of China, 2018) and *Determination of crude fat in feed* (GB/T 6433-2006) (China, Standardization Administration of the People’s Republic of China, 2006), respectively. Dry matter was analyzed according to the Chinese National Standards *Determination of moisture in feed* (GB/T 6435-2014) (China, Standardization Administration of the People’s Republic of China, 2014). Apparent digestibility was calculated based on the results of nutrient contents measurements.

Apparent digestibility (%) = [(total nutrient intake - total nutrient excretion)/total nutrient intake] × 100%.

#### Excreta microbiota analysis

At the end of the trial, fresh and uncontaminated excreta samples were collected from each replicate and stored in 2 mL cryotubes at −80°C for 16S rRNA sequencing. According to the manufacturer’s instructions, the total DNA was extracted from excreta samples using a Magnetic Soil and Stool DNA Kit (Tiangen Biotech Co., Ltd., Beijing, China; cat.# DP712). Briefly, 0.25–0.5 g of excreta sample was weighed, after which the following steps were performed in sequence: buffer SA, buffer SC, RNase A, and grinding beads were added; the mixture was vortexed and heated at 70°C to achieve bacterial lysis; and then centrifuged to collect the supernatant. Next, buffer SH was added to the supernatant, the mixture was incubated at 4°C for 10 min, and centrifuged to collect the supernatant again. Subsequently, isopropanol-containing GFA and magnetic bead suspension G were added to the supernatant collected in the previous step. The mixture was then oscillated for binding, followed by magnetic separation. The beads were then washed sequentially with protein removal solution (RD) and washing solution (PWD), and air-dried. DNA was eluted using elution buffer (TB) at 56°C. The DNA solution was subsequently collected after magnetic separation and stored at -80°C. The concentration and purity of extracted DNA were quantified using a Thermo NanoDrop One (Thermo Fisher Scientific, MA, United States). Subsequently, the V3–V4 hypervariable regions of the bacterial 16S rRNA gene were amplified using specific primers (forward: 5’-ACTCCTACGGGAGGCAGCA-3’; reverse: 5’-GGACTACHVGGGTWTCTAAT-3’). Library construction was carried out following the standard protocol of the ALFA-SEQ DNA Library Prep Kit, and the size of the library fragments was evaluated on the Qsep400 High-Throughput Nucleic Acid & Protein Analysis System (Hangzhou Houze Biotechnology Co., Ltd., China). The concentration of the library was measured using a Qubit4.0 (Thermo Fisher Scientific, Waltham, United States). Finally, the constructed amplicon libraries were subjected to PE250 sequencing on the Illumina Nova 6000 system (Guangdong Magigene Biotechnology Co., Ltd., Guangzhou, China).

The raw reads were quality-filtered by fastp (v0.14.1) (Chen et al., 2018). Concurrently, using cutadapt software (v1.9.1) and based on primer information at the 5’ and 3’ ends of sequences, primers were removed from the data, thus obtaining quality-controlled paired-end clean reads. Then the sequences were assembled by usearch-fastq_mergepairs (v10). Quality trimming of raw tags was performed using fastp (v0.14.1) with the parameters (-W 4 -M 20) to generate high-quality sequences. Across all 25 samples, a total of 2,777,908 high-quality sequences were obtained, with an average sequencing depth of 111116.32 sequences per sample. DADA2 was used for fast noise reduction and the generation of an ASV (Amplicon Sequence Variant) abundance table (Callahan et al., 2016). The ASVs inferred by DADA2 are hereafter referred to as operational taxonomic units (OTUs) throughout the text for consistency with the figure and table labels. Subsequently, all downstream bioinformatics analyses were performed within the QIIME 2 platform (Caporaso et al., 2010; Bolyen et al., 2019). The taxonomy of ASVs was analyzed by the QIIME2 feature-classifier classify-sklearn, based on the 16S rRNA database (Silva v138) (Bokulich et al., 2018). Functional potential of the gut microbiota was predicted using PICRUSt2 (version 2.5.0) based on 16S rRNA gene sequences obtained from excreta samples (Douglas et al., 2020). Stratified analysis was performed to generate ASV-level functional contributions, which were then aggregated to the genus level to assess the contribution of specific bacterial genera to KEGG pathways (Level 3). The predicted functions were annotated against the KEGG database. Differential abundance analysis of specific microbial taxa (phylum, genus) was performed using the Kruskal-Wallis test. Alpha diversity was assessed using the Chao1, Shannon, and Simpson indices, with significant differences among groups determined by the Kruskal-Wallis test. Beta diversity was calculated using the Bray-Curtis dissimilarity index and visualized through Principal Coordinate Analysis (PCoA) and non-metric multidimensional scaling (NMDS), while the analysis of similarity (ANOSIM) was conducted based on the Bray-Curtis distance matrix to determine differences in community composition.

#### Statistical analysis

Data on laying performance, egg quality, and nutrient apparent digestibility were analyzed using SPSS software (version 27.0). Normality and homogeneity of variances were confirmed using the Shapiro–Wilk and Levene’s tests, respectively, before performing a one-way ANOVA. Polynomial contrasts and Tukey’s HSD test were employed to identify dose-response trends and conduct all pairwise comparisons, respectively. Results are expressed as means and the pooled standard error of the mean (SEM). Analysis of microbial diversity and associated statistics was conducted in R (v4.4.3). The overall differences among groups were first evaluated using the Kruskal–Wallis test, followed by polynomial contrasts applied to rank-transformed relative abundance data to assess linear and quadratic trends. Spearman’s correlation analysis was performed to evaluate the relationships between excreta microbiota and production performance, as well as apparent nutrient digestibility. To control the false discovery rate (FDR), the Benjamini-Hochberg method was applied to the correlation *P*-values. An FDR-adjusted *P* < 0.05 was considered statistically significant. For all statistical analyses, the replicate served as the experimental unit. *P* < 0.05 was considered statistically significant.

## Results

### Production performance

The effects of dietary MTM on laying hen performance are presented in Table 2. During weeks 1–4, the laying rate responded both linearly (*P* = 0.034) and quadratically (*P* = 0.035) to incremental MTM additions. The MTM2.5 group achieved the highest laying rate (86.06%), which was significantly higher than that of the MTM7.5 and MTM10.0 groups during the first 4 weeks. However, no significant differences were observed during weeks 5–8 or overall (weeks 1–8) (*P* > 0.05). During the entire 1–8 week experimental period and each sub-phase (weeks 1–4 and 5–8), average egg weight differed significantly (*P* < 0.01) among treatments and showed significant linear (*P* < 0.05) and quadratic (*P* < 0.01) responses to dietary MTM levels. Throughout the trial period, the MTM5.0 group consistently yielded the highest average egg weight, significantly greater than all the other groups (*P* < 0.05), whereas the MTM10.0 group had the lowest. A linear trend in ADFI (*P* = 0.029 for weeks 5–8 and *P* = 0.015 for the overall period) was observed as MTM levels increased; however, the corresponding overall ANOVA F-tests were not significant (*P* = 0.142 and 0.112, respectively). Across all phases of the trial, MTM supplementation significantly affected FCR, with both significant linear and quadratic trends observed (*P* < 0.05). Specifically, the MTM5.0 group showed a significantly lower FCR than the higher-dose groups (MTM7.5 and MTM10.0) during weeks 1–4 and weeks 1–8 (*P* < 0.05), as well as during weeks 5–8, when compared with the MTM10.0 group (*P* < 0.05).

**TABLE 2:** Effect of dietary MTM supplementation on laying production performance.

Period	Items	Groups					SEM	*P*-value		
		CON	MTM2.5	MTM5.0	MTM7.5	MTM10.0		ANOVA	Linear	Quadratic
1–4 weeks	Laying rate, %	81.30^ac^	86.06^a^	84.73^ab^	78.72^c^	80.07^bc^	1.14	0.005	0.034	0.035
	Average egg weight, g	60.64^b^	60.32^b^	65.38^a^	60.29^b^	56.96^b^	1.11	< 0.001	0.048	< 0.001
	ADFI, g/d/hen	108.25	108.50	108.64	108.78	108.72	0.22	0.468	0.093	0.465
	Feed-egg ratio, g/g	2.25^ab^	2.13^b^	2.13^b^	2.33^a^	2.31^a^	0.04	0.002	0.013	0.013
5–8 weeks	Laying rate, %	79.64	75.48	76.99	78.92	77.38	2.83	0.839	0.901	0.589
	Average egg weight, g	60.49^b^	60.01^b^	67.02^a^	60.13^b^	53.56^c^	1.47	< 0.001	0.008	< 0.001
	ADFI, g/d/hen	108.50	108.42	108.84	109.09	108.92	0.20	0.142	0.029	0.656
	Feed-egg ratio, g/g	2.24^ab^	2.35^ab^	2.16^b^	2.26^ab^	2.40^a^	0.05	0.026	0.170	0.082
1–8 weeks	Laying rate, %	80.47	80.77	80.86	78.82	78.72	1.14	0.777	0.296	0.632
	Average egg weight, g	60.57^b^	60.17^b^	66.20^a^	60.21^b^	55.26^c^	1.10	< 0.001	0.006	< 0.001
	ADFI, g/d/hen	108.38	108.46	108.74	108.93	108.82	0.16	0.112	0.015	0.448
	Feed-egg ratio, g/g	2.25^ab^	2.24^ab^	2.14^b^	2.30^a^	2.35^a^	0.04	0.007	0.026	0.011

^a,b,c^Means within the same row with different superscripts are significantly different (*P* < 0.05). SEM, pooled standard error of the mean.

### Egg quality

The effects of dietary MTM supplementation on egg quality parameters are summarized in Table 3. A significant linear response to increasing MTM levels was detected in yolk color grade (*P* < 0.01). Compared with the CON group, yolk color grade was significantly increased in the MTM5.0, MTM7.5 and MTM10.0 groups (*P* < 0.05). However, the Haugh unit showed both significant linear (*P* = 0.020) and quadratic (*P* = 0.001) responses to rising MTM levels. The MTM5.0 group exhibited a significantly higher Haugh unit value than all the other treatments (*P* < 0.05). Yolk and albumen ratios displayed significant opposing linear trends in response to rising MTM levels, with yolk ratio increasing (*P* = 0.026) and albumen ratio decreasing (*P* = 0.020); however, one-way ANOVA revealed no significant differences in mean values among the treatments. No treatment effects were observed in albumen height, shell strength, and egg shape index (*P* > 0.05).

**TABLE 3:** Effect of dietary MTM supplementation on egg quality.

Items	Groups					SEM	*P*-value		
	CON	MTM2.5	MTM5.0	MTM7.5	MTM10.0		ANOVA	Linear	Quadratic
Egg weight, g	61.69	60.44	61.20	60.57	60.79	0.96	0.887	0.586	0.671
Albumen height, mm	4.05	4.27	4.19	4.07	4.04	0.22	0.927	0.745	0.521
Yolk color	6.27^b^	6.13^b^	7.23^a^	7.17^a^	7.23^a^	0.21	0.001	< 0.001	0.346
Haugh unit	72.37^b^	70.76^b^	79.75^a^	69.62^b^	67.57^b^	1.34	< 0.001	0.020	0.001
Eggshell strength, N	37.23	35.18	33.89	35.62	38.00	2.20	0.696	0.779	0.164
Shell ratio, %	12.90	12.96	13.12	12.82	13.29	0.29	0.797	0.493	0.740
Yolk ratio, %	27.43	27.15	28.13	28.17	28.56	0.43	0.169	0.026	0.806
Albumen ratio, %	59.67	59.89	58.75	59.00	58.15	0.49	0.127	0.020	0.682
Egg shape index	1.32	1.34	1.34	1.33	1.33	0.01	0.825	0.985	0.248

^a,b^Means within the same row with different superscripts are significantly different (*P* < 0.05). SEM, pooled standard error of the mean.

### Apparent nutrient digestibility

A significant effect of treatment was observed on the apparent crude protein digestibility (*P* = 0.018, Table 4). The MTM5.0 group showed significantly higher apparent crude protein digestibility compared with all the other treatment groups (*P* < 0.05). In contrast, the apparent digestibility of dry matter and crude fat, as well as the metabolizability of gross energy, were not significantly affected by dietary MTM supplementation (*P* > 0.05, Table 4).

**TABLE 4:** Effect of dietary MTM supplementation on nutrient apparent digestibility and energy metabolizability.

Items	Groups					SEM	*P*-value		
	CON	MTM2.5	MTM5	MTM7.5	MTM10.0		ANOVA	Linear	Quadratic
Gross energy, %	70.74	70.82	72.35	71.08	72.61	0.745	0.261	0.105	0.978
Dry matter, %	66.41	66.66	67.37	66.60	67.57	0.777	0.781	0.369	0.982
Crude protein, %	56.43^b^	54.37^b^	63.32^a^	57.45^b^	57.85^b^	1.70	0.018	0.285	0.135
Crude fat, %	82.03	81.74	79.64	79.01	81.85	1.573	0.532	0.543	0.203

^a,b^Means within the same row with different superscripts are significantly different (*P* < 0.05). SEM: pooled standard error of the mean.

### Excreta microbiota

**FIGURE 1:**
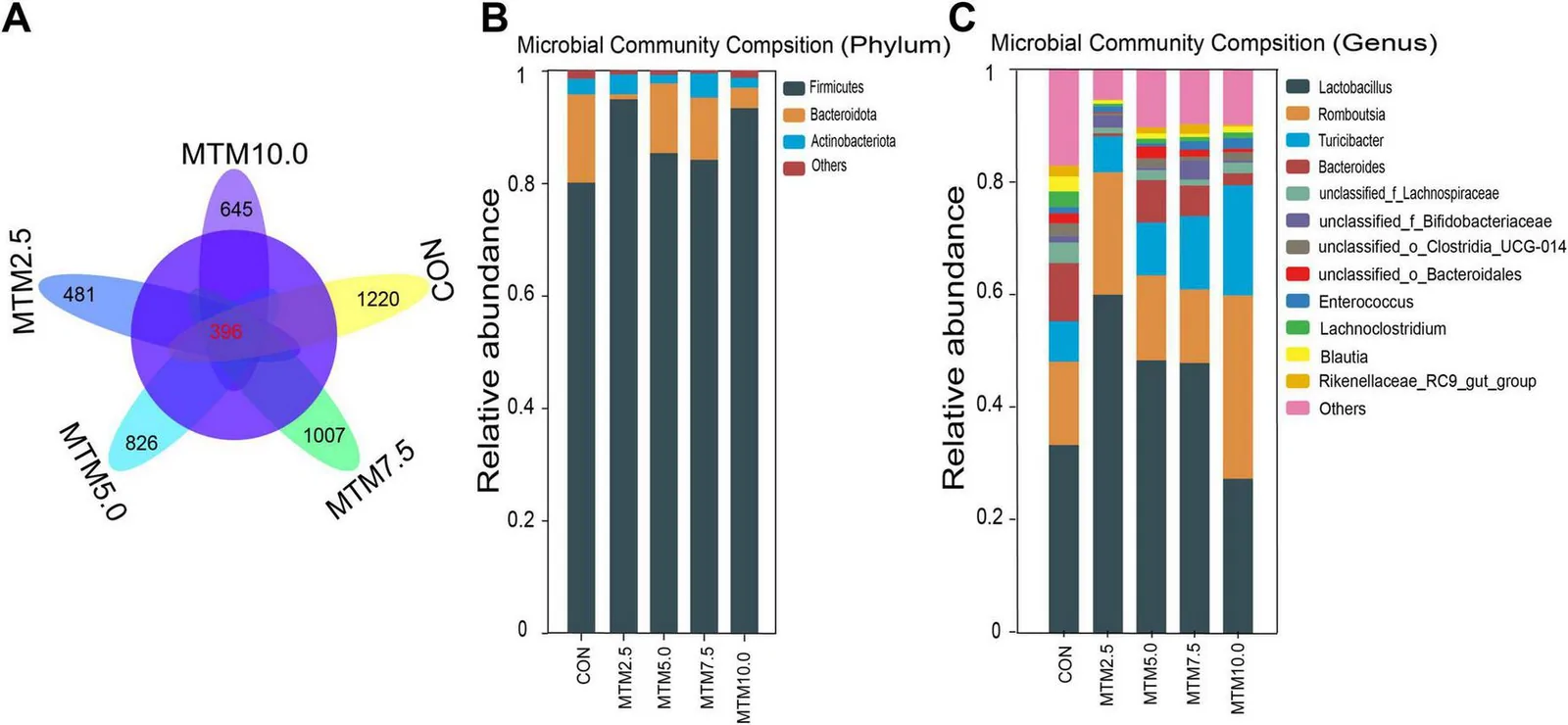
Composition of excreta microbiota in late-phase laying hens. **(A)** Venn diagram illustrating all shared and unique OTUs identified in the excreta microbiota among groups. **(B)** Relative abundance of the top bacterial phyla. **(C)** Relative abundance of the top bacterial genera. The overall community composition at the phylum and genus levels did not differ significantly among groups (Kruskal-Wallis test, *P* ≥ 0.05). However, polynomial contrast analysis revealed significant quadratic trends for *Bacteroidota* (phylum) and *Bacteroides* (genus), a near-significant quadratic trend for *Lactobacillus*, and a significant linear increase for *Turicibacter* with increasing MTM levels (see Table 5 for statistical details).

16S rRNA gene sequencing was performed to characterize the composition of the excreta microbiota. The Venn diagram visualized the distribution of shared and unique OTUs among the groups (Figure 1A). A total of 396 bacterial OTUs were shared among all groups, while 1,220, 481, 826, 1,007, and 645 unique OTUs were identified in excreta samples of laying hens from the CON, MTM2.5, MTM5.0, MTM7.5, and MTM10.0 groups, respectively. Analysis of species composition showed that at the phylum level, three phyla (*Firmicutes*, *Bacteroidota*, and *Actinobacteriota*) dominated the microbial communities, with *Firmicutes* being the most abundant (Figure 1B). At the genus level, eight predominant microbial genera (*Lactobacillus*, *Romboutsia*, *Turicibacter*, *Bacteroides*, *Enterococcus*, *Lachnoclostridium*, *Blautia*, and *Rikenellaceae_RC9_gut_group*) were identified, with *Lactobacillus* exhibiting the highest relative abundance (Figure 1C).

**FIGURE 2:**
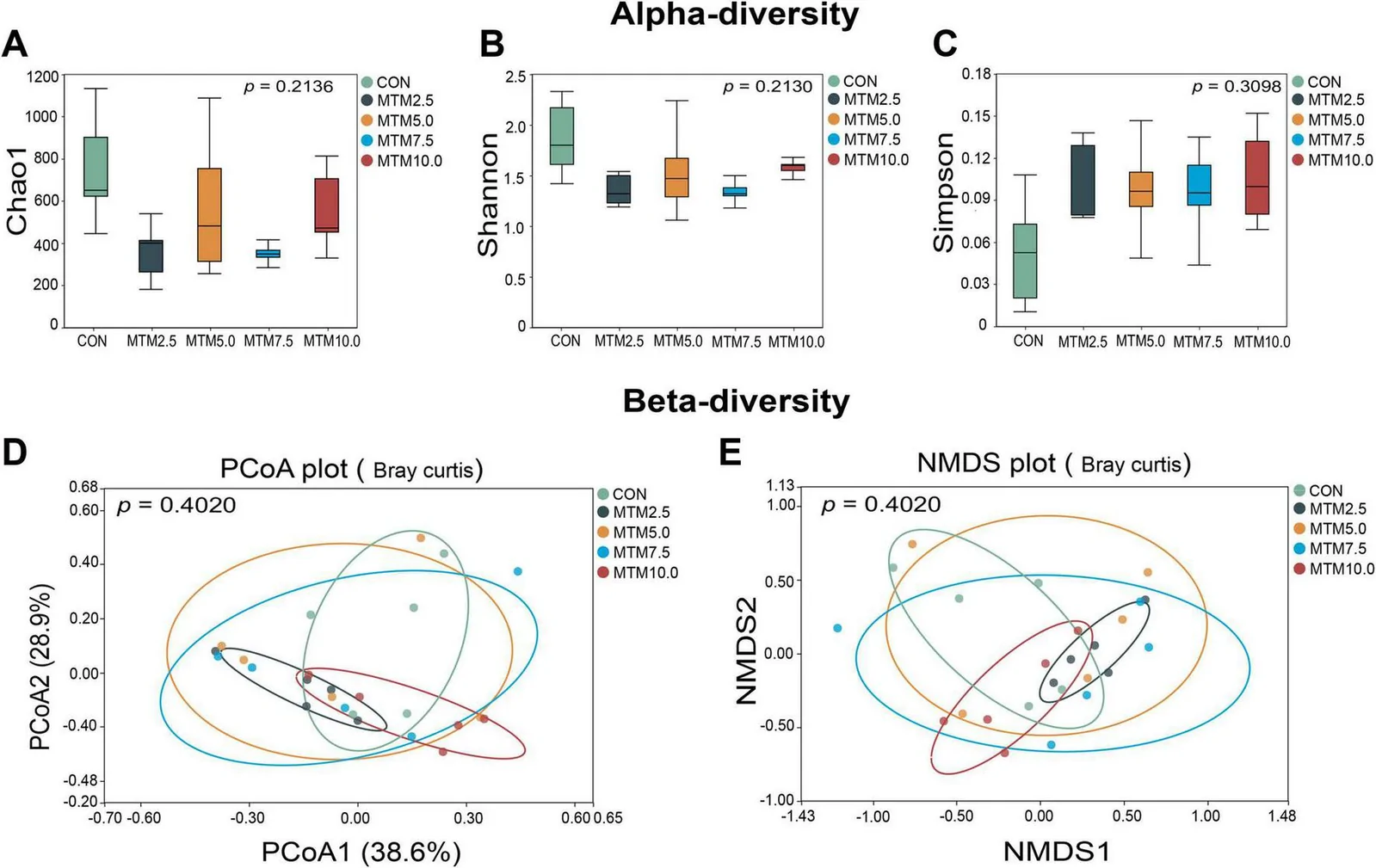
Alpha (α)- and beta (β)-diversity patterns of the excreta microbiota in late-phase laying hens. **(A–C)** Alpha-diversity indices (Chao1, Shannon, and Simpson). The Kruskal-Wallis test detected no significant differences among groups (*P* > 0.05). **(D,E)** Beta-diversity assessed by principal coordinates analysis (PCoA) and non-metric multidimensional scaling (NMDS) plots based on Bray-Curtis distances. No significant separation was observed among groups (*P* > 0.05).

Following the differential abundance analysis, no significant differences in overall community composition were observed at either the phylum or genus level. However, significant quadratic responses to MTM inclusion levels were observed for the relative abundances of the phylum *Bacteroidota* (*P* = 0.047) and the genus *Bacteroides* (*P* = 0.031), with both decreasing in the MTM2.5 and MTM10.0 groups but remaining at control levels in the MTM5.0 and MTM7.5 groups (Figures 1B,C and Table 5). The relative abundance of *Lactobacillus* showed a tendency toward significance in its quadratic trend (*P* = 0.064), peaking at MTM2.5 and then declining in the MTM5.0 and MTM7.5 groups relative to the CON group, with the MTM10.0 group dropping below the CON group level (Figure 1C and Table 5). Furthermore, *Turicibacter* exhibited a significant linear increase in relative abundance with rising MTM supplementation levels (*P* = 0.049, Figure 1C and Table 5). No significant differences were observed in either alpha or beta diversity across the groups (*P* > 0.05; Figure 2).

**TABLE 5:** Effect of dietary MTM supplementation on the relative abundance of excreta microbiota.

Microbiota taxon	Groups					SEM	*P*-value		
	CON	MTM2.5	MTM5	MTM7.5	MTM10.0		K-W *P*-value^1^	Linear^2^	Quadratic^3^
Phylum									
Firmicutes	0.804	0.951	0.859	0.852	0.935	0.074	0.330	0.233	0.206
Bacteroidota	0.154	0.008	0.119	0.103	0.036	0.074	0.084	0.445	0.047
Actinobacteriota	0.027	0.035	0.014	0.040	0.017	0.012	0.369	0.139	0.661
Genus									
Lactobacillus	0.339	0.615	0.511	0.502	0.304	0.116	0.302	0.670	0.064
Romboutsia	0.152	0.213	0.143	0.127	0.309	0.052	0.203	0.324	0.187
Turicibacter	0.070	0.059	0.085	0.122	0.183	0.037	0.216	0.049	0.199
Bacteroides	0.101	0.004	0.073	0.052	0.021	0.046	0.082	0.483	0.031

^1^K-W *P*-value: *P*-value from the Kruskal-Wallis test among all experimental groups.

^2^Linear: *P*-value for the test of a linear trend across MTM levels, performed on rank-transformed relative abundance data.

^3^Quadratic: *P*-value for the test of a quadratic trend across MTM levels, performed on rank-transformed relative abundance data.

**FIGURE 3:**
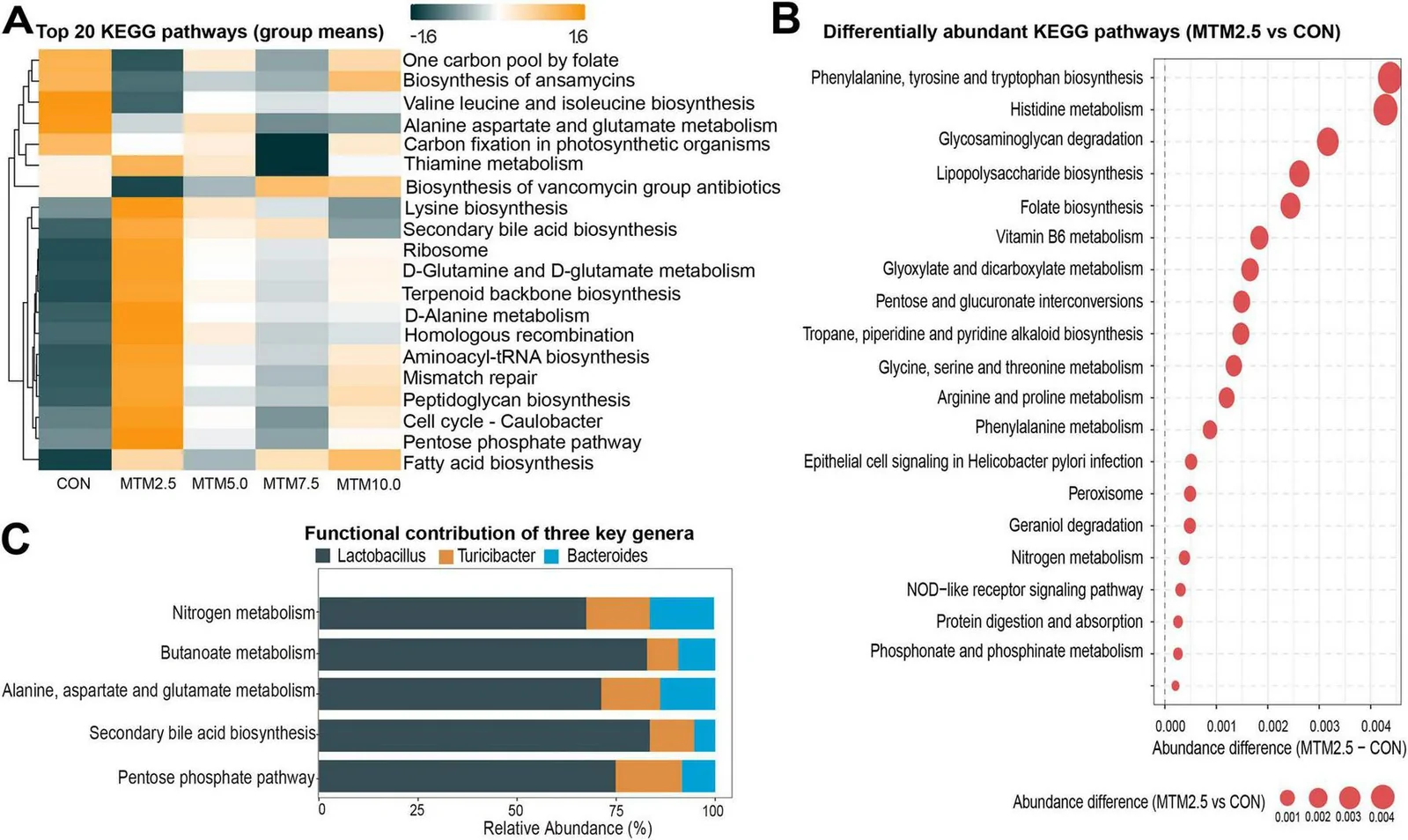
Functional pathways associated with MTM supplementation and contribution of key genera. **(A)** Heatmap of the top 20 KEGG pathways across five treatment groups. **(B)** Dot plot of 19 differentially abundant pathways between the MTM2.5 and CON groups (global permutation test *P* = 0.045). The *X*-axis shows the abundance difference (MTM2.5 vs. CON), and point size reflects the magnitude of difference. All 19 pathways were upregulated in the MTM2.5 group, primarily involving amino acid, carbohydrate, and lipid metabolism. **(C)** Relative contribution (%) of selected key genera (*Bacteroides*, *Lactobacillus*, and *Turicibacter*) to five representative KEGG pathways, based on normalized abundance from PICRUSt2 stratified analysis.

To further elucidate the functional implications of MTM-modulated microbiota, PICRUSt2-based functional prediction was performed. The heatmap of the top 20 KEGG pathways revealed that the CON group showed lower abundance across most pathways, while the MTM2.5 group exhibited the highest abundance; the MTM5.0, MTM7.5, and MTM10.0 groups displayed intermediate levels with similar abundance patterns (Figure 3A). Pairwise comparisons between each MTM dose and CON suggested that the 2.5% MTM dose exhibited the most pronounced functional alterations (Figure 3B), with 19 KEGG pathways significantly upregulated (global permutation test *P* = 0.045), predominantly involved in amino acid, lipid, carbohydrate, and energy metabolism. Functional contribution analysis further suggested that Bacteroides showed relatively higher contributions to nitrogen metabolism and the pentose phosphate pathway; Lactobacillus exhibited consistently high contributions across all five pathways, with the highest contributions to butanoate metabolism and secondary bile acid biosynthesis; while Turicibacter showed similar contributions to nitrogen metabolism, the pentose phosphate pathway, and alanine/aspartate/glutamate metabolism, with the lowest contribution to butanoate metabolism (Figure 3C).

Correlation analysis was performed to examine the associations of excreta microbiota with production performance and nutrient utilization. As shown in Table 6, at the genus level, the relative abundance of *Turicibacter* showed a negative correlation with average egg weight (*R* = −0.407, *P* = 0.045), although this correlation was not statistically significant after FDR correction (adjusted *P* = 0.404). No other significant correlations were observed between the assessed microbial taxa and the production performance or the nutrient metabolism parameters.

**TABLE 6:** Correlation of excreta microbiota with production performance (1–8 weeks) and nutrient utilization.^1,2,3^

Microbiota taxon	Laying rate		Average egg weight		ADFI		FCR		Gross energy		Dry matter		Crude protein		Crude fat	
	*R*	*P*	*R*	*P*	*R*	*P*	*R*	*P*	*R*	*P*	*R*	*P*	*R*	*P*	*R*	*P*
Phylum																
Firmicutes	−0.162	0.439	0.072	0.731	0.112	0.595	−0.058	0.782	0.030	0.887	−0.195	0.348	−0.140	0.503	−0.324	0.115
Bacteroidota	0.204	0.327	−0.105	0.615	−0.043	0.837	0.025	0.904	0.058	0.781	0.168	0.421	0.204	0.327	0.295	0.153
Actinobacteriota	0.200	0.336	−0.165	0.428	−0.204	0.328	0.276	0.182	−0.263	0.203	0.022	0.919	−0.023	0.914	0.080	0.703
Genus																
Lactobacillus	−0.015	0.943	0.218	0.294	−0.214	0.304	−0.100	0.634	−0.138	0.510	−0.245	0.238	0.021	0.922	−0.203	0.329
Romboutsia	0.008	0.972	−0.277	0.180	0.170	0.416	0.116	0.582	−0.034	0.873	−0.167	0.423	−0.257	0.214	−0.142	0.498
Turicibacter	0.162	0.439	−0.407	0.045	0.151	0.470	0.250	0.229	−0.035	0.870	−0.040	0.850	−0.113	0.589	−0.002	0.993
Bacteroides	0.164	0.432	−0.155	0.457	−0.022	0.915	0.053	0.801	0.037	0.861	0.155	0.459	0.164	0.432	0.254	0.220

^1^Correlation analysis was performed using Spearman’s rank correlation method (based on the data from 1 to 8 weeks). | R| values close to 1 indicate a strong monotonic relationship. R: Spearman’s rank correlation coefficient. P: *P*-value for the Spearman’s correlation test.

^2^FDR correction (Benjamini-Hochberg) was applied to production traits. No correlations remained significant after adjustment (all *q* > 0.05). The *Turicibacter*-egg weight correlation: raw *P* = 0.045; adjusted *P* = 0.404.

^3^All correlations should be interpreted as suggestive and require validation in larger cohorts.

## Discussion

The present study evaluated the effects of dietary MTM supplementation on production performance, egg quality, nutrient digestibility, and excreta microbiota composition in late-phase laying hens, revealing the dose-dependent effects. We demonstrated that 5.0% MTM was optimal for most metrics in laying hens, while supra-optimal MTM supplementation adversely affected production performance.

Our results demonstrated that dietary inclusion of 5.0% MTM significantly improved the average egg weight and the FCR in laying hens. These findings corroborate and extend previous reports on the efficacy of both silymarin and MTM in poultry nutrition (Hashemi Jabali et al., 2018; Guo et al., 2024; Khan et al., 2024). Notably, the increase in average egg weight observed in the MTM5.0 group was achieved without a significant increase in feed intake, suggesting enhanced metabolic efficiency rather than simply greater nutrient consumption. This observation aligns with the previously proposed mechanism that the bioactive compounds in MTM, particularly silymarin, may function by optimizing intestinal health and nutrient utilization (Hashemi Jabali et al., 2018; Guo et al., 2024). While previous studies have established the benefits of silymarin in various forms and doses, our study specifically identifies 5.0% MTM as an effective dietary level for late-phase laying hens, a population that has received limited attention in prior MTM research. The consistent performance improvement at this inclusion rate supports the potential of MTM as a valuable functional feed ingredient for sustaining productivity during the critical late laying period.

Notably, dietary MTM supplementation exerts dose-dependent and phase-specific effects on the production performance of laying hens. The average egg weight showed a consistent and significant quadratic response to increasing MTM levels throughout the entire 8-week experiment, peaking at 5.0% MTM and hitting its lowest point at 10.0%. The FCR also exhibited a quadratic relationship with dietary MTM levels, improving to an optimal value at 5.0% inclusion, beyond which further supplementation was detrimental. This dose-response pattern confirms that MTM’s benefits are constrained to a specific range, which aligns with the finding that 3% MTM yielded the best FCR (Hashemi Jabali et al., 2018). The laying rate during the early phase (weeks 1–4) also exhibited a significant quadratic response to MTM supplementation, with the MTM2.5 group achieving the highest laying rate and the laying rate declining gradually at higher inclusion levels. Interestingly, during weeks 5–8, the MTM2.5 and MTM5.0 groups exhibited a more pronounced reduction in laying rate compared with the control, MTM7.5, and MTM10.0 groups. Laying performance is determined by the development of follicles in the ovary (Ru et al., 2024; Bai et al., 2026), a process strictly regulated by the precise positive and negative feedback mechanisms of the hypothalamic-pituitary-gonadal (HPG) axis (Zhao et al., 2023; Cheng et al., 2025). Therefore, in addition to the typical age-related decline in the late production period, the initial laying-rate improvement from low-to-moderate MTM (2.5, 5.0%) might be related to overstimulation of the reproductive axis. This could potentially trigger compensatory depletion and physiological adaptation, which ultimately may have exacerbated the subsequent decline. However, we acknowledge that this interpretation remains speculative and requires further validation. Future studies examining HPG axis gene expression, plasma gonadotropin and sex steroid levels, and follicular development are needed to test this hypothesis and to elucidate the potential mechanisms through which MTM regulates the laying rate via the HPG axis.

In addition to production performance, egg quality was also evaluated, with yolk color and Haugh unit being the key indicators. This study demonstrated that dietary MTM significantly improved yolk color score, which is consistent with previous reports that dietary silymarin supplementation improved egg yolk color in laying hens (Ahammad et al., 2024a; Khan et al., 2024; Gousias et al., 2025). This may be attributed to its ability to enhance pigments deposition in egg yolk while protecting pigment from oxidation. Within the hen, carotenoids undergo enzymatic changes (such as oxidation and cleavage) before being deposited in the yolk, which ultimately determines the yolk color (Dansou et al., 2023). Silymarin demonstrates potent antioxidant capacity across a broad spectrum of physiological conditions and disease states in both humans and various animal species (Wang et al., 2020; Bahari et al., 2024; Sharma et al., 2025). Furthermore, research has demonstrated that silymarin promotes lipid metabolism by regulating the expression of key hepatic genes, including ACC, FASN, and SREBP-1 (Guo et al., 2024). Therefore, MTM can enhance hepatic function and promote lipid metabolism via its silymarin component, while concurrently protecting pigments (e.g., carotenoids) from oxidative degradation during absorption and transport processes. This dual action promotes the formation and yolk-directed translocation of pigment-lipid complexes, ultimately increasing pigment deposition rates and leading to improved yolk color.

The Haugh unit, is the primary indicator for assessing egg albumen quality and freshness (Sarlak et al., 2021). Established as an industry standard for evaluating egg quality by the U.S. poultry industry in 1937 (Gabriela da Silva Pires et al., 2020), it is primarily determined by the viscosity of the thick albumen in the egg, with higher values indicating better albumen quality and freshness. We observed that dietary inclusion of 2.5 and 5.0% MTM significantly increased the Haugh unit values of eggs, which is consistent with the improvements reported with 30 g/kg MTM supplementation (Gholamalian et al., 2022). In addition, dietary milk thistle powder at 1.5% has been found to elevate Haugh unit values in Japanese quail eggs (Rivas-Caceres et al., 2024). Furthermore, prior studies have confirmed that dietary silymarin supplementation increased Haugh unit values of hen eggs (Ahammad et al., 2024a; Ahammad et al., 2024b). Oxidative stress diminishes albumen viscosity and reduces Haugh unit values by increasing proteolysis, whereas dietary supplementation with plant-derived natural antioxidants enhances radical-scavenging activity in the albumen and increases Haugh unit values (Obianwuna et al., 2022). As previously mentioned, silymarin exhibits antioxidant properties; therefore, dietary MTM could enhance protein metabolism and utilization by alleviating oxidative stress, subsequently improving Haugh unit values. This finding is further supported by the increased apparent crude protein digestibility observed in the MTM5.0 group.

To shed light on the mechanisms through which dietary MTM affects the production performance and egg quality of laying hens, we characterized excreta microbiota via 16S rRNA sequencing to assess the gut microbial composition. The gut microbiota plays an important role in regulating immune function, nutrient metabolism, and intestinal homeostasis (Khan et al., 2020; Kogut et al., 2020; Rychlik, 2020). We propose that by fostering beneficial bacteria and inhibiting detrimental ones, dietary MTM modulates the gut microbiota, thereby improving intestinal health and subsequent production performance. This proposed mechanism is well supported by multiple studies investigating both MTM and its bioactive compounds. For instance, supplementing laying hen diets with 3% MTM significantly reduced ileal *E. coli* counts and achieved the optimal FCR (Hashemi Jabali et al., 2018). Similarly, silymarin supplementation linearly increased the population of *Lactobacillus* and decreased those of *Escherichia coli* in broilers (Shanmugam et al., 2022). Moreover, in broilers challenged with either *E. coli* or aflatoxins, dietary silymarin supplementation improved growth performance by suppressing ileal pathogenic microorganisms, including *Escherichia coli*, *Salmonella*, *Klebsiella*, and total Gram-negative bacteria (Jahanian et al., 2017; Jahanian et al., 2021).

In the current study, the excreta microbiota of laying hens were dominated by *Firmicutes* and *Bacteroidota* at the phylum level, with *Lactobacillus, Romboutsia*, *Bacteroides*, and *Turicibacter* being the dominant genera, consistent with prior findings (Hamid et al., 2019; Khan et al., 2020; Chen et al., 2023). Despite the absence of statistically significant differences in overall microbial community composition and in the relative abundance of specific taxa, some discernible trends were observed. We found significant quadratic responses to the inclusion of MTM levels in the relative abundances of the phylum *Bacteroidota* and the genus *Bacteroides*. The 2.5 and 10.0% MTM groups exhibited lower relative abundances of the phylum *Bacteroidota* and the genus *Bacteroides* than the CON group, whereas the 5.0 and 7.5% groups maintained levels similar to the CON group. *Bacteroidota*, particularly the genus *Bacteroides*, encode a large number of genes involved in carbohydrate metabolism and participate in its regulation (Medvecky et al., 2018; Pudlo et al., 2022). In addition, Bacteroides is a key degrader of complex carbohydrates in the chicken gut, with higher abundance linked to increased short-chain fatty acid production and reduced gut inflammation markers (Fan et al., 2023), further supporting its role in nutrient metabolism. Furthermore, we found that the relative abundance of *Lactobacillus* increased in the low- and medium-dose MTM groups, peaking in the MTM2.5 group. Subsequently, it declined with increasing MTM doses and ultimately dropped below the level of the CON group in the MTM10.0 group. Studies indicate that *Lactobacillus* contributes to the maintenance of intestinal microecological health in chickens and the enhancement of FCR (Wang et al., 2017; Yan et al., 2017). Additionally, it has been reported that an increased relative abundance of *Lactobacillus* in the gut was associated with enhanced laying rate and feed intake in laying hens (Qiao et al., 2019). Notably, with increasing MTM dose, we observed a linear increase in the relative abundance of *Turicibacter*, which was negatively correlated with egg weight. Although this correlation did not remain significant after FDR correction, the trend is consistent with prior reports that *Turicibacter* is negatively associated with laying rate and egg weight (Gan et al., 2020). It has been reported that *Turicibacter* can modulate lipid metabolism through bile salt hydrolase-mediated bile acid deconjugation (Lynch et al., 2023). Furthermore, dietary supplementation with 60 mg/kg bile acids has been demonstrated to improve the laying rate and FCR in hens (Yang et al., 2022). Moreover, dietary bile acid supplementation ameliorates hepatic lipid metabolism and increases both laying rate and feed intake in hens (Xing et al., 2025). Therefore, MTM might regulate lipid metabolism through *Turicibacter*, ultimately affecting egg weight.

To further elucidate the functional implications of MTM-modulated microbiota, we performed PICRUSt2-based functional prediction. The 2.5% MTM dose significantly upregulated 19 KEGG pathways, primarily involved in amino acid, carbohydrate, and lipid metabolism, with functional alterations most pronounced at this dose, whereas the 5.0, 7.5, and 10.0% doses showed similarly attenuated responses, suggesting a non-linear dose-dependent relationship between MTM and microbial function. Functional contribution analysis further revealed that *Lactobacillus* consistently exhibited the highest contributions across all five pathways, particularly in butanoate metabolism and secondary bile acid biosynthesis, indicating its broad metabolic versatility (Widodo et al., 2021). *Bacteroides* showed relatively higher contributions to nitrogen metabolism and the pentose phosphate pathway, consistent with its role in nitrogen assimilation and carbohydrate metabolism (Gardner and Schreier, 2021). while *Turicibacter* showed higher contribution in secondary bile acid biosynthesis than *Bacteroides*, but its contributions were substantially lower than those of *Lactobacillus* across all pathways, with the lowest contribution in butanoate metabolism, suggesting its primary involvement in energy and amino acid metabolism rather than short-chain fatty acid synthesis (Lynch et al., 2023). The upregulation of amino acid metabolism pathways at the 2.5% dose aligned with the trend toward improved crude protein digestibility, and the upregulation of lipid metabolism pathways may have provided a metabolic basis for improved yolk color. However, pathway activation preceded the phenotypic appearance (which became significant at ≥ 5.0% dose), suggesting that sustained functional modulation may be required for MTM’s effects on yolk color to manifest. Collectively, these findings indicate that MTM-induced functional remodeling is dose-dependent and genus-specific, with Lactobacillus playing a central role in multiple metabolic pathways.

Collectively, dietary MTM did not significantly alter overall microbial community composition at any inclusion level. However, polynomial contrast analysis revealed non-linear (quadratic) dose-response relationships for the phylum *Bacteroidota* and its representative genus *Bacteroides*, a near-significant quadratic trend for *Lactobacillus*, and a significant linear increase in *Turicibacter* with increasing MTM levels. Functional prediction using PICRUSt2 identified the most pronounced pathway upregulation at the 2.5% dose (19 KEGG pathways) compared with the control, primarily involving amino acid, carbohydrate, and lipid metabolism, suggesting that MTM may influence specific taxa and their metabolic functions in a dose-dependent manner. However, given the absence of significant compositional differences and the application of FDR correction for multiple comparisons (after which the *Turicibacter*–egg weight correlation did not remain significant), the mechanistic link between MTM, microbiota, and performance remains correlational and requires further validation.

**FIGURE 4:**
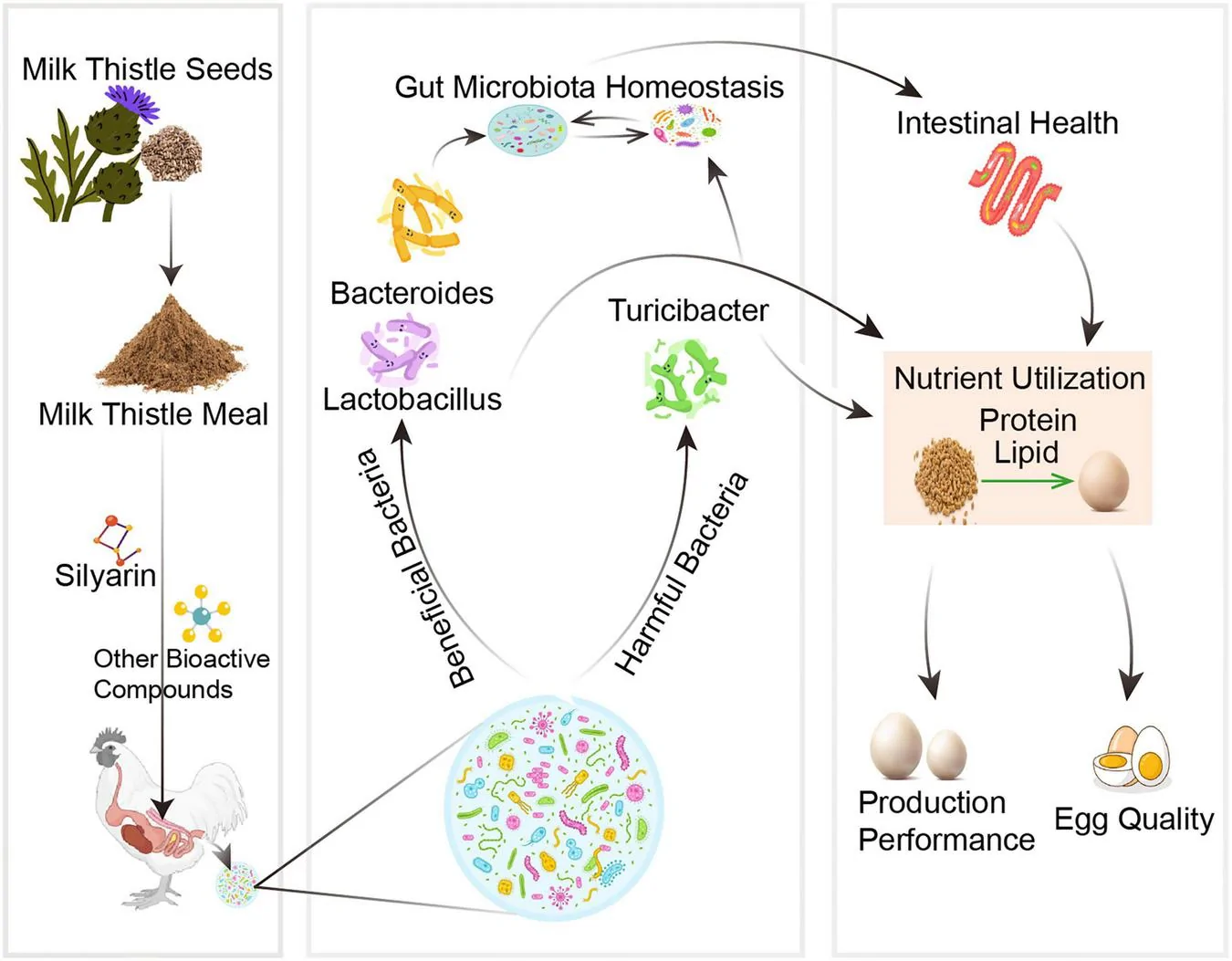
Hypothetical mechanism of dietary Milk Thistle Meal (MTM) in late-phase laying hens. Based on the present findings and previous literature, dietary inclusion of MTM is proposed to modulate specific microbial taxa (e.g., *Lactobacillus*, *Bacteroides* and *Turicibacter*), which in turn may influence nutrient utilization and ultimately contribute to improved production performance (e.g., increased average egg weight and lower FCR) and superior egg quality (e.g., higher Haugh unit and darker yolk color). However, these mechanistic links are correlational and hypothetical, and require direct experimental validation.

In summary, as outlined in the hypothetical model (Figure 4), dietary MTM was associated with improved production performance and egg quality in late-laying hens, alongside enhanced nutrient utilization. The model integrates these findings with the observed microbial trends and proposes potential links to intestinal health and metabolism. However, we acknowledge that these mechanistic links were not experimentally validated in the present study (e.g., through assessments of intestinal morphology, barrier integrity, inflammatory responses, microbial metabolites, or nutrient transporter expression) and therefore remain hypothetical. Although these results are encouraging, further research is warranted to confirm long-term applicability and elucidate the underlying mechanisms. Overall, MTM represents a promising feed additive for sustainable poultry production.

## Conclusion

This study demonstrates the feasibility of incorporating milk thistle meal (MTM) as an unconventional feed ingredient in late-laying hen diets. Dietary 5.0% MTM improved production performance and egg quality, alongside enhanced crude protein digestibility and suggestive changes in specific microbial taxa. However, given the absence of significant compositional differences, the mechanistic link between MTM, microbiota, and performance remains correlational and requires further validation. These results support MTM as a sustainable feed resource and warrant further validation under commercial conditions.

## Data Availability

All raw FASTQ sequencing files generated in this study have been deposited in Figshare (https://doi.org/10.6084/m9.figshare.32778159). For further inquiries, please contact the corresponding authors.
